# Afternoon exercise was associated with greater fat loss than morning exercise in overweight/obese adults: an exploratory study of PER3 rs228697 genotype matching

**DOI:** 10.3389/fphys.2026.1872652

**Published:** 2026-07-30

**Authors:** Yuchen Wang, Chunyan Xu, Juan Tong

**Affiliations:** 1 Department of Sport Science Institute, Beijing Sport University, Beijing, China; 2 Beijing Higher School Engineering Research Center of Sport Nutrition, Beijing Sport University, Beijing, China; 3 National Institute of Sports Medicine, General Administration of Sport of China, Beijing, China

**Keywords:** body composition, circadian rhythm, exercise timing, moderate-intensity continuous training, obesity, PER3 rs228697

## Abstract

**Objective:**

Exercise timing may influence metabolic responses and body composition, but the extent to which circadian genetic variation contributes remains unclear. The PER3 rs228697 polymorphism has been linked to chronotype-related traits, although its relevance to exercise timing remains uncertain. This exploratory study examined whether body-composition responses to morning versus afternoon moderate-intensity continuous training (MICT) differed according to PER3 rs228697 genotype in overweight/obese university students.

**Methods:**

In this 8-week supervised intervention, 31 overweight/obese university students performed MICT in the morning (7:00–10:00) or afternoon (16:00–19:00). Participants with the CC genotype were allocated to a morning training group (CC-AM) or an afternoon training group (CC-PM), and G-allele carriers were included in an afternoon group (G-PM). Body weight, body mass index (BMI), waist circumference, body fat percentage, total fat mass, and regional fat mass were assessed before and after the intervention. Circadian preference, physical activity, sleep quality, and fatigue were also evaluated. Because the G-PM subgroup was very small, findings for this subgroup were treated as exploratory.

**Results:**

Across the overall sample, body weight, BMI, waist circumference, body fat percentage, and total fat mass decreased over the 8-week intervention. In descriptive subgroup analyses, the CC-PM group showed larger reductions in several body-composition outcomes, including body weight, BMI, waist circumference, body fat percentage, and total fat mass, than the CC-AM group. A similar pattern was observed for several regional fat measures, particularly trunk, android, and gynoid fat mass. By contrast, findings for G-allele carriers were difficult to interpret because of the very small subgroup size. Estimated between-group differences were generally modest and imprecise, and behavioral chronotype did not clearly correspond with PER3 genotype.

**Conclusions:**

In this exploratory sample of overweight/obese university students, 8 weeks of MICT was associated with favorable changes in several body-composition outcomes. Among CC genotype carriers, afternoon training was associated with descriptively greater improvements than morning training; however, the estimates were imprecise and do not support strong conclusions regarding genotype-based exercise-time matching based on PER3 rs228697 alone. Larger, well-controlled studies with objective circadian phenotyping are needed to clarify the potential role of genotype-informed exercise timing in personalized weight management.

## Introduction

Circadian rhythms regulate a broad range of physiological processes, including energy metabolism, hormone secretion, sleep–wake cycles, and exercise performance (Pi-Sunyer, 2009; Froy, 2010; Bray et al., 2017; Noh, 2018). Increasing evidence suggests that exercise is not only an effective lifestyle strategy for improving metabolic health but also a non-photic zeitgeber capable of modulating peripheral circadian clocks. Consequently, the timing of exercise may influence metabolic responses to training as well as the effectiveness of fat-loss interventions, making exercise timing an important topic in both circadian biology and exercise science (Delezie and Challet, 2011; Summa and Turek, 2014; Kanikowska et al., 2015; Kim et al., 2023; Lan et al., 2025a). However, current evidence regarding the effects of exercising at different times of day on weight-loss outcomes remains inconsistent (Stoner et al., 2016). In a randomized controlled trial involving 88 overweight and obese young adults, Willis et al. found that participants assigned to morning exercise achieved significantly greater reductions in body weight and fat mass over 10 months than those assigned to afternoon exercise (Willis et al., 2020a). Other studies, however, have reported different results. Savikj et al. showed that afternoon high-intensity interval exercise was more effective than morning exercise in lowering blood glucose levels in patients with type 2 diabetes (Savikj et al., 2019). Arciero et al. reported that morning exercise was particularly beneficial for reducing abdominal fat, whereas evening exercise was more effective in improving muscle performance in women (Arciero et al., 2022). In addition, Creasy et al. and Mancilla et al. found that afternoon or evening exercise may offer certain advantages for weight loss, fat reduction, and metabolic improvement (Mancilla et al., 2021; Creasy et al., 2022). Taken together, these inconsistent findings suggest that the effects of exercise timing may be moderated by individual differences rather than determined by a single “optimal” time for all individuals (Vitale and Weydahl, 2017; Arciero et al., 2022). In addition to inter-individual variability, differences in exercise intensity and training modality may also contribute to the heterogeneous findings reported in the literature, as the time of day most favorable for exercise may depend on the physiological demands of the exercise performed (Chtourou and Souissi, 2012a). For example, higher-intensity exercise may be more advantageous in the afternoon or evening, whereas lower-intensity exercise may be better tolerated or more effective in the morning (Teo et al., 2011; Lan et al., 2025b). It is therefore important to investigate exercise timing from the perspective of both individual variability and exercise characteristics.

Genetic factors, particularly polymorphisms in circadian rhythm-related genes, may represent one of the key mechanisms underlying these differences (Katzenberg et al., 1998; Hida et al., 2014a). Several circadian clock genes, including CLOCK, ARNTL/BMAL1, CRY, and PER family genes, have been implicated in chronotype- and sleep-related phenotypes. Among these candidate genes, PER3 (period circadian regulator 3) has been extensively studied in relation to chronotype, sleep timing, and behavioral rhythmicity. Previous studies suggest that PER3 polymorphisms may influence an individual’s adaptation to circadian disruption and may thereby contribute to variation in metabolic phenotypes and behavioral rhythm patterns (Archer et al., 2003a; Archer et al., 2003b; Archer et al., 2018). The rs228697 polymorphism is a common variant within the PER3 gene and has been reported to be associated with chronotype tendency, with the G allele more frequently linked to evening preference and the C allele to morning preference (Hida et al., 2014a; Hida et al., 2014b; Liberman et al., 2017). Although this association has not been entirely consistent across studies, it nevertheless supports the use of this variant as a candidate marker for exploring the relationship between genetic circadian traits and exercise timing. Given the exploratory nature of the present study and the limited sample size, we focused on this prespecified candidate locus rather than examining a broader panel of circadian gene variants.

Although an increasing number of studies have examined the effects of exercise performed at different times of day on weight control and body composition, their findings remain inconsistent (Stoner et al., 2016). Moreover, most previous studies have focused on average group-level effects and have paid limited attention to inter-individual differences in circadian traits and genetic background. Research examining whether PER3 polymorphisms modulate weight-loss responses to exercise performed at different times of day remains scarce, particularly intervention studies integrating genotype, behavioral rhythmicity, and exercise timing. This gap has limited the development of personalized exercise-timing strategies for precision weight management. Against this background, the present study focused on the PER3 rs228697 polymorphism and developed an exploratory genotype–exercise timing matching model to deliver an 8-week moderate-intensity continuous training (MICT) intervention in overweight and obese university students. MICT was chosen because it is a practical and commonly recommended exercise modality for weight management, is generally well tolerated in overweight populations, and allows relatively standardized control of exercise intensity and volume. However, the effects of exercise timing may differ under other exercise intensities or modalities, such as high-intensity interval training or resistance training, which may evoke different physiological and perceptual responses. Therefore, the present findings should be interpreted as specific to MICT. By comparing changes in body weight, body fat, and regional body composition between genotype-matched and genotype-mismatched exercise timing groups, this study aimed to examine the potential interaction between genotype and exercise timing and to provide preliminary evidence for personalized exercise interventions based on circadian and genetic characteristics.

## Methods

### Participants

Based on previous evidence linking the PER3 rs228697 polymorphism to circadian preference, an exploratory genotype–exercise timing matching model was developed in this study. The inclusion criteria were as follows (Pi-Sunyer, 2009): overweight or obesity, defined as a body fat percentage >25% in men and >30% in women (Gallagher et al., 2000) (Froy, 2010); a sedentary lifestyle or low physical activity level, defined as <150 minutes of moderate-intensity exercise per week; and (Bray et al., 2017) regular sleep–wake patterns, based on self-reported fixed bedtimes and wake times with only minor fluctuations of up to ±1 hour. The exclusion criteria were as follows (Pi-Sunyer, 2009): diseases likely to substantially affect metabolism or exercise capacity, such as diabetes, thyroid dysfunction, or cardiovascular disease (Froy, 2010); sleep disorders, such as insomnia or obstructive sleep apnea; and (Bray et al., 2017) current use of medications known to influence circadian timing or sleep and thereby potentially affect study outcomes, such as thyroid hormones, melatonin, or glucocorticoids. A total of 40 overweight or obese university students were screened, of whom 35 met the inclusion criteria and completed baseline assessments. Ultimately, 31 participants completed the 8-week intervention and were included in the final analysis. The study protocol was approved by the Ethics Committee of Beijing Sport University (approval no. [2025438H]), and all participants provided written informed consent before participation.

Based on previous evidence suggesting an association between the PER3 rs228697 polymorphism and chronotype tendency, the C allele was considered, for exploratory purposes, to reflect a relatively stronger morning tendency, whereas the G allele was considered to reflect a relatively stronger evening tendency. Accordingly, an exploratory genotype–exercise timing matching model was constructed. Morning exercise performed by participants with the CC genotype was defined as genotype-matched exercise timing (CC-AM group), whereas afternoon exercise performed by participants with the CC genotype was defined as genotype-mismatched exercise timing (CC-PM group). Because the G allele occurs at a relatively low frequency (G = 0.05935 in East Asian populations) (Auton et al., 2015; Karczewski et al., 2020), resulting in a limited number of carriers in the present sample, G-allele carriers were assigned to afternoon exercise as an exploratory matched group (G-PM group). It should be emphasized that this matching framework was exploratory in nature and was intended to provide a preliminary assessment of the potential relationship between genetic circadian traits and exercise timing rather than a definitive classification of biological matching.

### Study design

**Figure 1:**
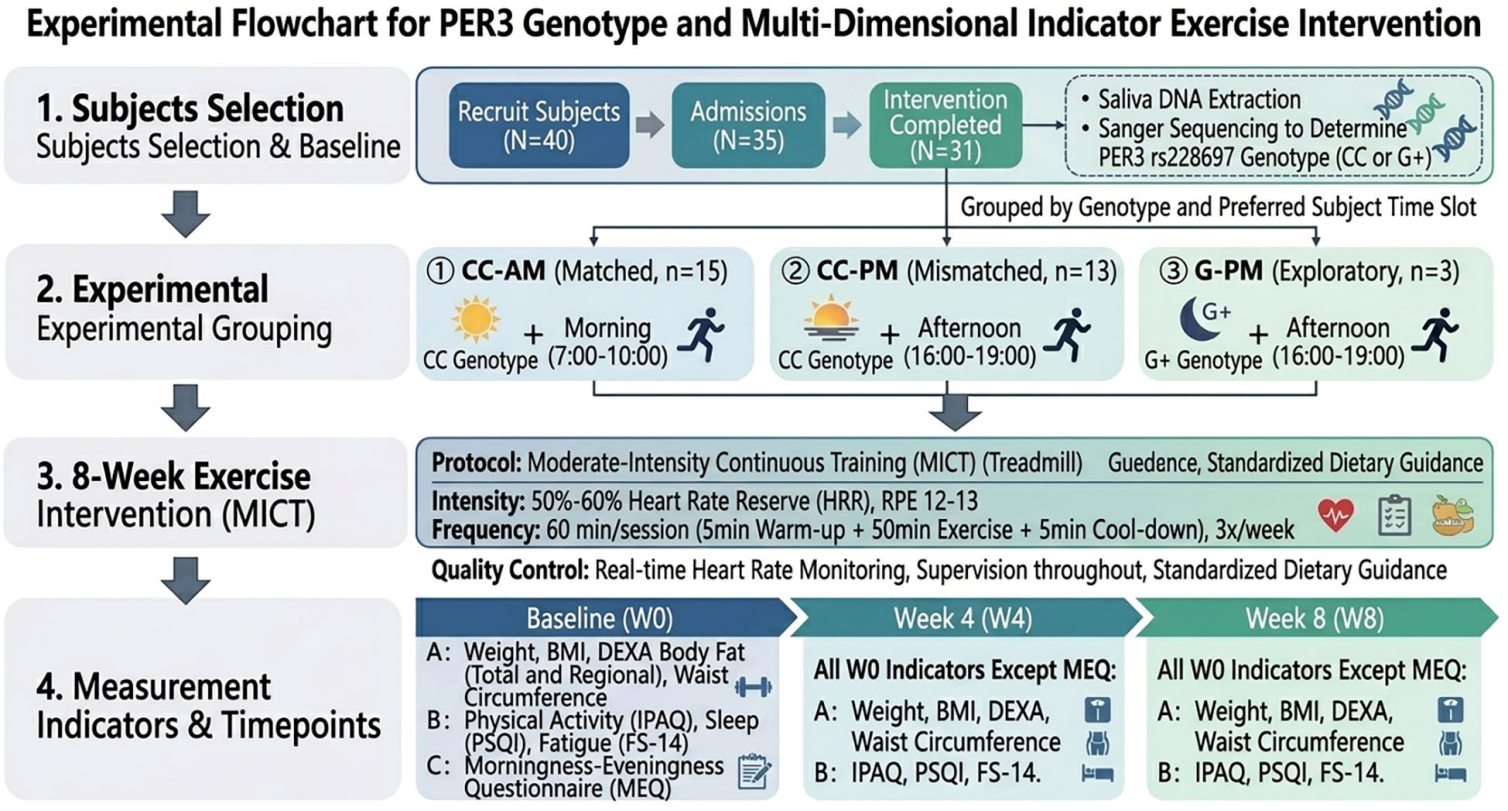
Flowchart.

The intervention lasted 8 weeks and consisted of an MICT protocol, primarily treadmill-based, performed at 50%–60% of heart rate reserve (HRR), corresponding to a rating of perceived exertion (RPE) of 12–13 (Borg, 1982). Each session lasted 60 minutes, including a 5-minute warm-up, 50 minutes of main exercise, and a 5-minute cool-down. Training was performed three times per week, with at least 24 hours between sessions (Garber et al., 2011). Morning exercise sessions were scheduled between 7:00 and 10:00, and afternoon sessions between 16:00 and 19:00, consistent with commonly used time-of-day categories in exercise timing research (Savikj et al., 2019). All participants were instructed to complete training within the assigned time window, and any deviation exceeding 10 minutes was recorded as a timing-adherence deviation. To minimize the influence of training-load differences on the outcomes, all sessions were supervised, and exercise intensity was monitored in real time using an arm-strap heart rate monitor. For verification of training intensity, heart rate data were retrieved from the device records, and the mean heart rate during the 50-min main exercise phase was calculated for each session. These values were averaged across the three weekly sessions and then summarized over the 8-week intervention for comparison between the morning and afternoon groups. To reduce the influence of lifestyle-related confounding factors, participants were instructed to maintain their habitual diet and daily activity patterns throughout the intervention and to refrain from participating in any additional structured weight-loss training programs. Physical activity level (Qu and Li, 2004; Hagströmer et al., 2008), sleep quality (Buysse et al., 1989; Liu and Tang, 1996), and fatigue (Chalder et al., 1993; Tang et al., 2022) were assessed using questionnaires at baseline and again at weeks 4 and 8, and were taken into account when interpreting the findings. A flowchart of participant recruitment and group allocation is shown in **Figure 1**.

### Assessment of lifestyle-related factors during the intervention

To evaluate potential lifestyle-related influences on intervention outcomes, physical activity, sleep quality, fatigue, and self-reported sleep–wake schedules were assessed during the intervention. Physical activity was evaluated using the International Physical Activity Questionnaire (IPAQ), and the following component scores were extracted: walking time, moderate-intensity activity, vigorous-intensity activity, total physical activity (MET), and sitting time. IPAQ assessments were performed at baseline, week 2, week 4, week 6, and week 8 to characterize self-reported physical activity patterns over the course of the intervention. Sleep quality and fatigue were assessed using the Pittsburgh Sleep Quality Index (PSQI) and the Fatigue Scale-14 (FS-14), respectively. In addition, self-reported bedtime and wake-up time were recorded at baseline, week 4, and week 8 to describe habitual sleep–wake schedules.

### Genotype analysis

**Figure 2:**
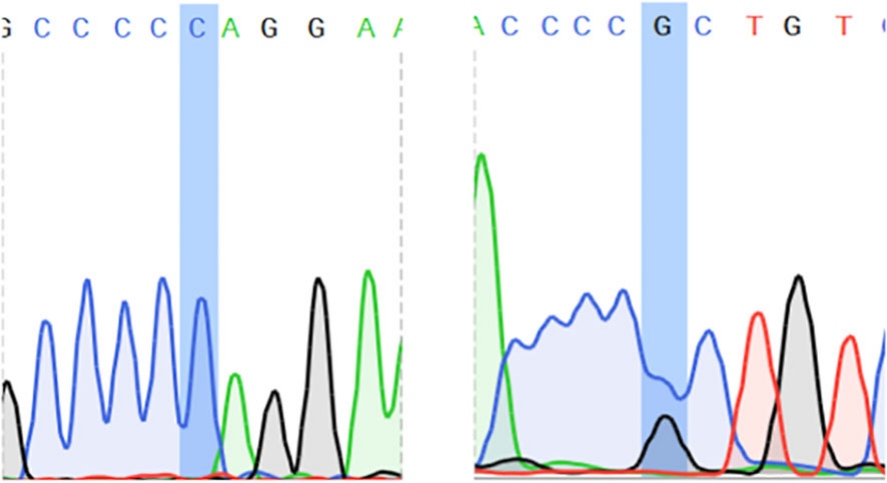
Genotyping results of the PER3 rs228697 locus (left: CC genotype; right: CG genotype).

Saliva samples were collected from each participant for PER3 rs228697 genotyping. DNA was extracted and purified using the TIANamp Swab DNA Kit. The purified DNA was amplified using the following primers: forward, 5′-GCCTTACCCAGCTTTCCCTT-3′, and reverse, 5′-GCCTCCCACTTTTCCTCCTC-3′. After purification, the PCR products were analyzed by Sanger sequencing to determine each participant’s genotype at rs228697 (**Figure 2**).

### Anthropometric and body composition measurements

All assessments were conducted after a 4-hour fast. Body weight and body composition were measured using dual-energy X-ray absorptiometry (DEXA; GE Lunar Prodigy, USA). The main outcome measures included total body fat mass, body fat percentage, and regional fat mass in the upper limbs, thighs, trunk, android region, and gynoid region. Waist circumference was measured with a non-elastic tape at the midpoint between the highest point of the iliac crest and the lower border of the 12th rib, with participants standing naturally and at the end of a normal exhalation, to the nearest 0.1 cm.

### Circadian phenotype

Because genotype does not necessarily correspond fully to actual behavioral circadian phenotype, participants’ circadian preference was further assessed using the Morningness–Eveningness Questionnaire (MEQ) (Horne and Ostberg, 1976; Zhang et al., 2006). The conventional MEQ categories were used descriptively to characterize morning-, intermediate-, and evening-type tendency. However, because only a small number of participants in the present sample met the conventional criteria for definite morning type or evening type, additional exploratory subgroup analyses were based on the relative distribution of MEQ scores within the sample. Specifically, MEQ scores were ranked from highest to lowest, and participants in the upper third were categorized as the morning-leaning group, those in the lower third as the evening-leaning group, and those in the middle third as the intermediate group (Anderson et al., 2018a). This sample-based grouping approach was used to obtain more balanced subgroup sizes for exploratory comparisons of body-composition changes following the intervention.

### Statistical analysis

Statistical analyses were performed using SPSS 26.0. Continuous variables are presented as mean ± standard deviation (SD). Given the exploratory nature of the study and the limited sample size, assumptions of normality were not assessed solely by formal hypothesis tests. Instead, data distributions were evaluated using visual inspection of Q–Q plots and histograms, and homogeneity of variance was examined where appropriate to guide the choice of analytical methods. Within-group comparisons were performed using paired-samples t tests or the corresponding non-parametric tests, whereas between-group comparisons were conducted using independent-samples t tests or the corresponding non-parametric tests. To examine the interaction between group and time, mixed-design repeated-measures analysis of variance (ANOVA) was performed. *Post hoc* comparisons were adjusted using the Bonferroni method when necessary, and effect sizes were reported. Change scores were calculated as post − pre for each outcome. Independent-samples t-tests were used to compare change scores between the CC-PM and CC-AM groups (reference: CC-PM − CC-AM), with mean differences reported alongside 95% confidence intervals. In addition, exploratory descriptive comparisons were performed for changes in body weight, body fat percentage, and total fat mass across MEQ-defined chronotype categories (morning type, intermediate type, and evening type), with results summarized as mean differences and 95% confidence intervals. Given the exploratory nature of the study and the limited sample size, findings were interpreted by considering the magnitude, direction, and precision of the estimated effects rather than relying solely on a dichotomous significance threshold. Because of the very small sample size in the G-PM group, results from that group were treated as exploratory, and the primary analysis focused on comparisons between the CC-AM and CC-PM groups. For the two main analytic groups (CC-AM and CC-PM), IPAQ component scores were summarized across baseline, week 2, week 4, week 6, and week 8, and temporal patterns were examined using a 2 (group) × 5 (activity) × 5 (time) repeated-measures ANOVA. PSQI and FS-14 scores, as well as bedtime and wake-up time, were analyzed longitudinally to determine whether lifestyle-related factors changed during the intervention period.

## Results

A total of 35 overweight or obese university students were enrolled, and 31 completed the 8-week intervention and were included in the final analysis: 15 in the CC-AM group, 13 in the CC-PM group, and 3 in the G-PM group. Because the G allele is relatively uncommon in East Asian populations, the number of G-allele carriers in this sample was limited. Accordingly, this study should be regarded as exploratory overall, with findings involving G-allele carriers being especially preliminary and descriptive. Hardy–Weinberg equilibrium testing indicated no obvious departure from equilibrium at the PER3 rs228697 locus in this sample (χ² = 0.08, df = 1, P = 0.78). Baseline demographic and body-composition characteristics for the main analytic groups (CC-AM and CC-PM) are presented descriptively in **Table 1**. Descriptive data for the G-PM subgroup are also provided for completeness, but no inferential interpretation was based on this very small subgroup.

**Table 1:** Baseline body composition characteristics of participants in each group (x ± s).

Group	Age	Height (cm)	Weight (kg)	BMI(kg/m²)	Waist (cm)	Body fat percentage(%)	Fat mass(g)
CC-AM	20.6 ± 1.5	169.0 ± 8.2	65.44 ± 8.09	22.76 ± 3.22	75.71 ± 11.54	32.47 ± 3.81	21222.47 ± 4675.03
CC-PM	21.4 ± 2.5	165.9 ± 5.8	65.75 ± 12.64	23.71 ± 3.38	76.81 ± 11.21	32.22 ± 3.61	21139.92 ± 4991.54
G-PM	22.7 ± 1.7	172.3 ± 4.5	73.9 ± 11.97	24.76 ± 2.56	82.2 ± 7.19	29.4 ± 1.3	21513 ± 4235.95

The G-PM subgroup (n = 3) is shown descriptively for completeness only and was not included in the main inferential comparisons.

### Training intensity verification

Heart-rate data retrieved from the training records indicated that the prescribed MICT intensity was achieved similarly in the two main groups. The mean heart rate during the 50-min main exercise phase, averaged across the three weekly sessions and summarized over the 8-week intervention, was 129.2 ± 6.8 bpm in the morning group and 131.5 ± 7.3 bpm in the afternoon group. The proportion of exercise time spent within the target heart-rate zone was 72.4 ± 10.6% and 76.8 ± 12.1%, respectively. These values suggest that internal training load was broadly similar between groups.

### Effects of MICT on body composition

Across the overall sample, favorable pre-post changes were observed over the 8-week intervention. Mean body weight changed by −1.04 kg (95% CI, −1.72 to −0.36), BMI by −0.35 kg/m² (95% CI, −0.59 to −0.12), waist circumference by −2.27 cm (95% CI, −3.30 to −1.24), body fat percentage by −0.56% (95% CI, −1.06 to −0.05), and total body fat mass by −719 g (95% CI, −1259.36 to −178.63). Overall, these estimates were consistent with modest improvements in body-composition outcomes during the intervention.

### Effects of exercise timing within genotype-defined groups

**Figure 3:**
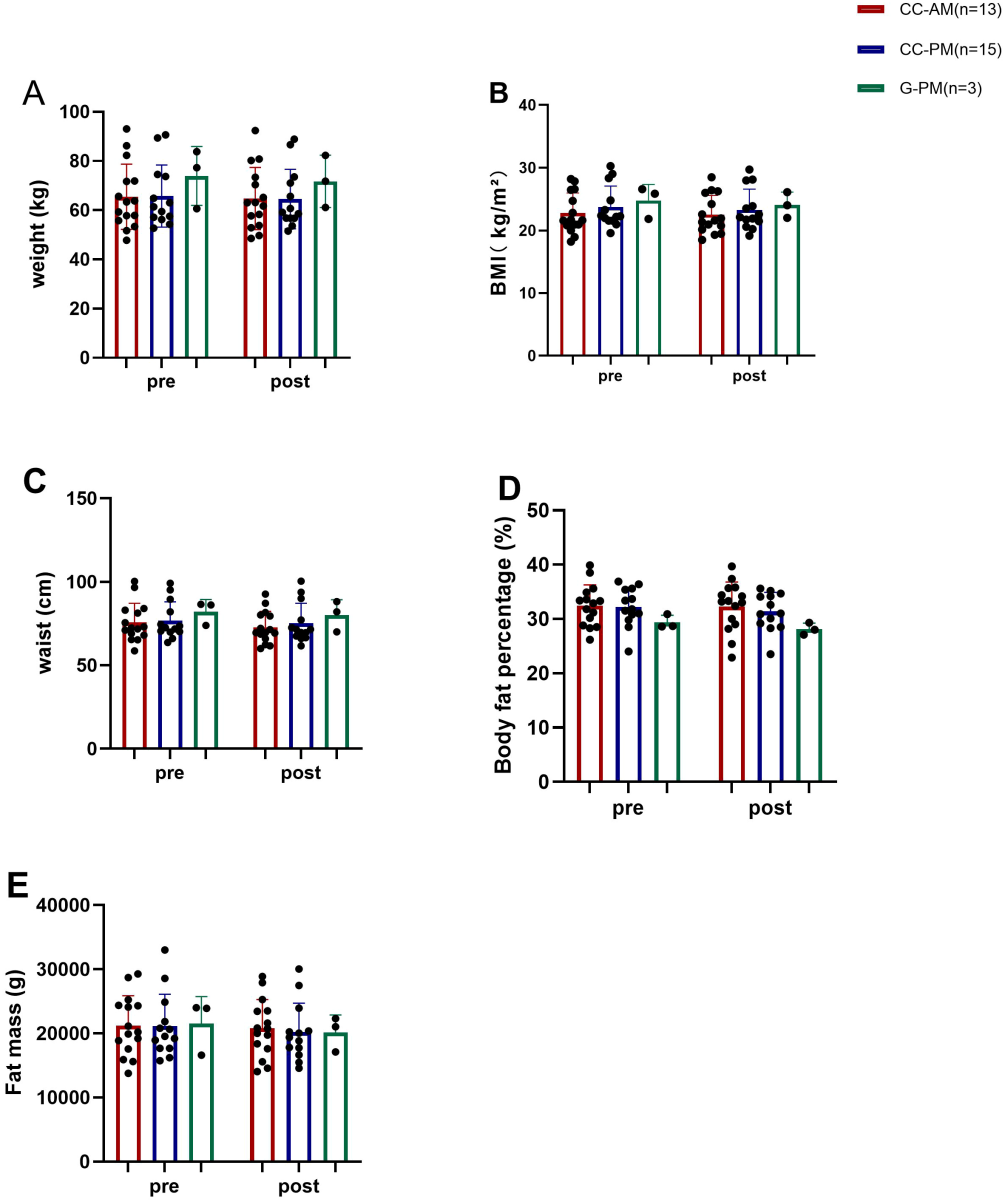
Individual pre- and post-intervention values for **(A)** Body weight, **(B)** BMI, **(C)** Waist circumference, **(D)** Body fat percentage, **(E)** Total fat mass across the three groups. Bars represent group means; error bars indicate standard deviations (SD); jittered points show individual data. Group sizes: n = 15 (CC-AM), 13 (CC-PM), 3 (G-PM). Mean changes with 95% confidence intervals are reported in the Results section.

**Figure 4:**
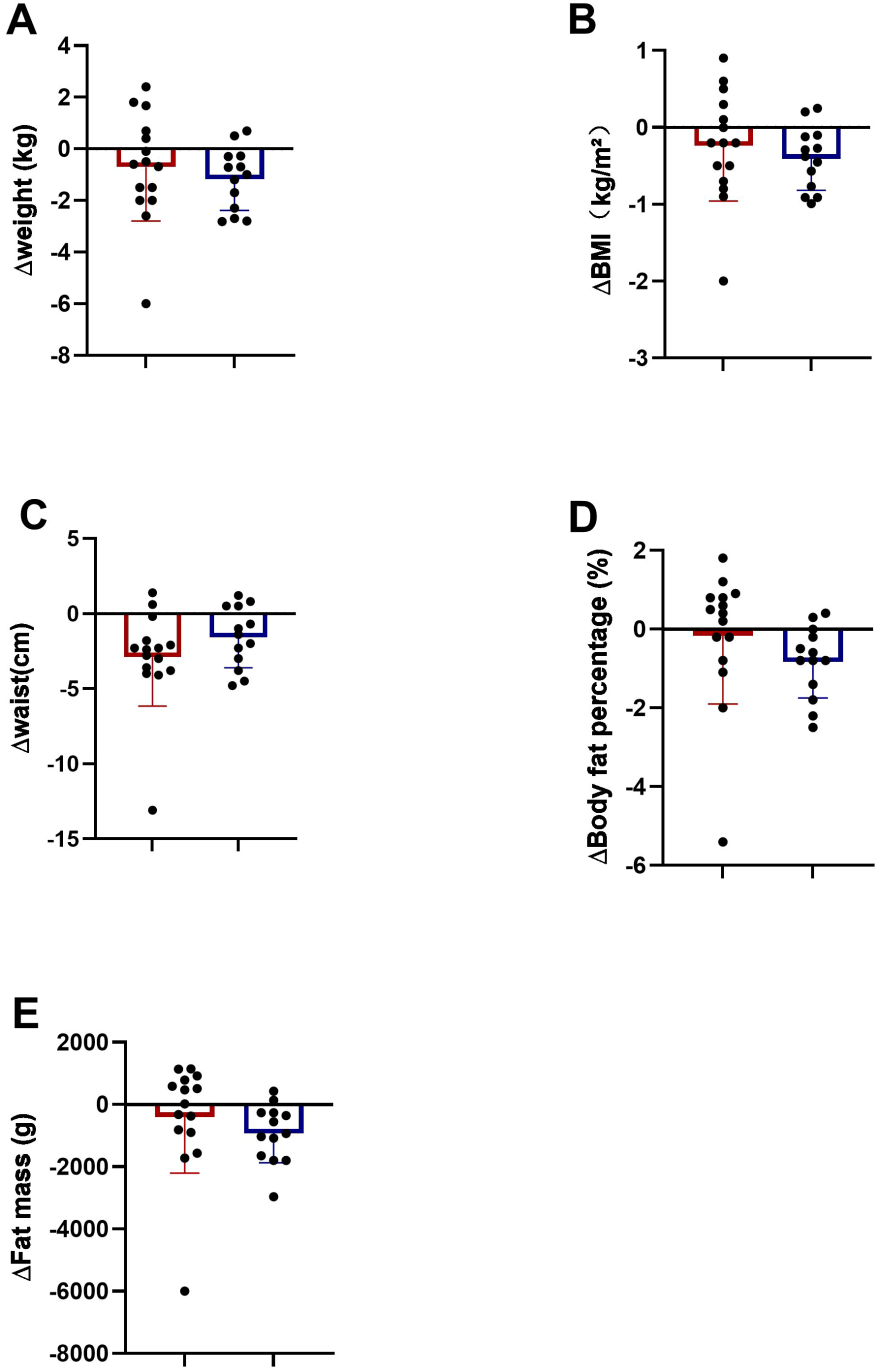
Change scores (post − pre) for **(A)** Body weight, **(B)** BMI, **(C)** Waist circumference, **(D)** Body fat percentage, **(E)** Total fat mass in the CC-AM and CC-PM groups. See **Figure 3** legend for details on data presentation. Group sizes: n = 15 (CC-AM), 13 (CC-PM), 3 (G-PM).

In descriptive subgroup analyses, the CC-PM group showed reductions in body weight (mean change = −1.18 kg, 95% CI, −1.91 to −0.45), BMI (mean change = −0.41 kg/m², 95% CI, −0.65 to −0.16), body fat percentage (mean change = −0.84%, 95% CI, −1.39 to −0.29), total body fat mass (mean change = −932 g, 95% CI, −1497.71 to −366.29), and waist circumference (mean change = −1.58 cm, 95% CI, −2.81 to −0.35). In the CC-AM group, waist circumference decreased by −2.90 cm (95% CI, −4.71 to −1.09), whereas the remaining outcomes also tended to decline, although the estimates were less precise (**Figure 3**). Comparisons of change scores suggested larger reductions in body weight, BMI, body fat percentage, and total fat mass in the CC-PM group than in the CC-AM group (**Figure 4**). However, the corresponding confidence intervals were wide, indicating limited precision.

**Figure 5:**
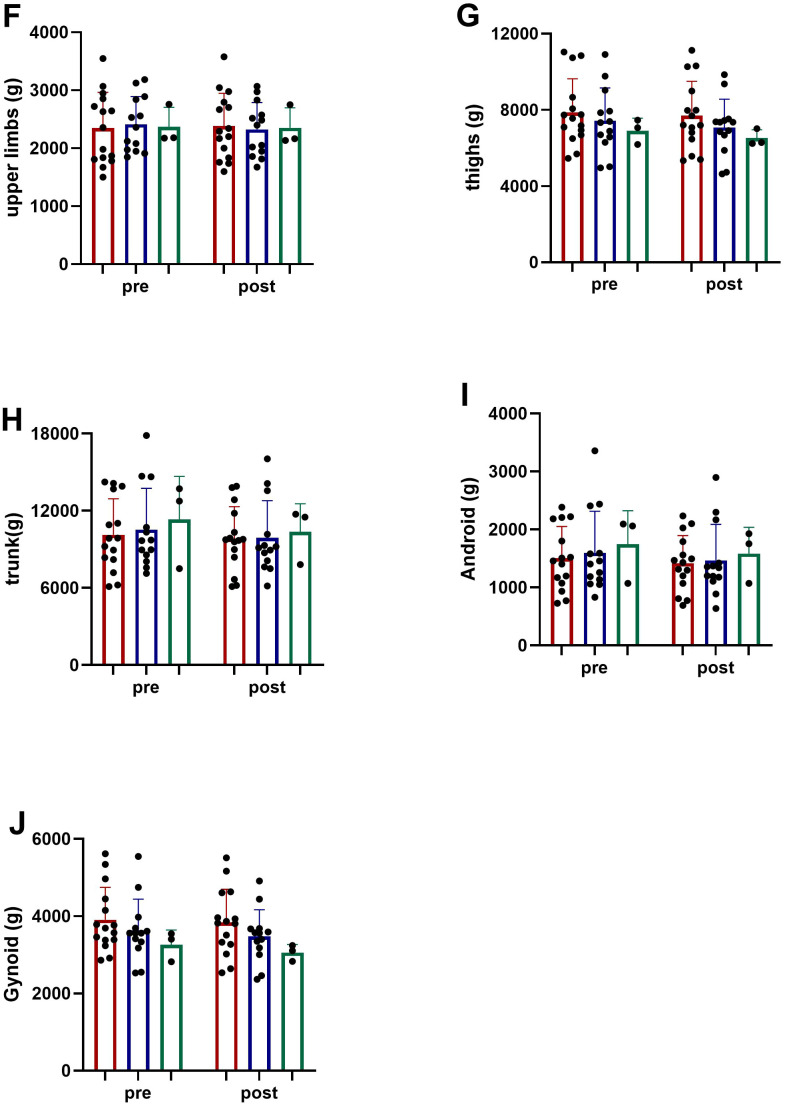
Individual pre- and post-intervention values for regional fat mass **(F)** Upper limbs, **(G)** Thighs, **(H)** Trunk, **(I)** Android region, **(J)** Gynoid region across the three groups. See **Figure 3** legend for details on data presentation. Group sizes: n = 15 (CC-AM), 13 (CC-PM), 3 (G-PM).

**Figure 6:**
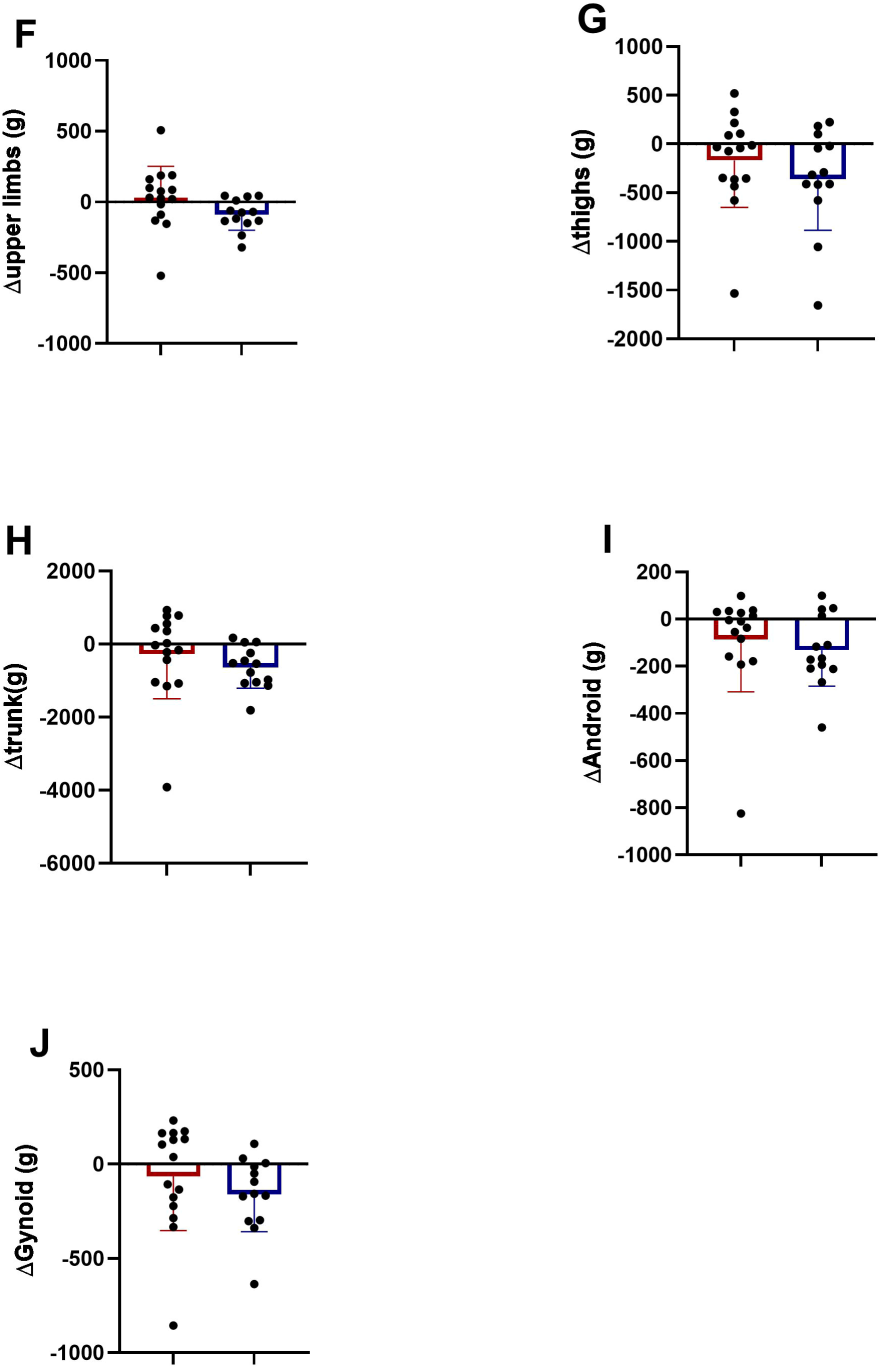
Change scores (post − pre) for regional fat mass **(F)** Upper limbs, **(G)** Thighs, **(H)** Trunk, **(I)** Android region, **(J)** Gynoid region in the CC-AM and CC-PM groups. See **Figure 3** legend for details on data presentation. Group sizes: n = 15 (CC-AM), 13 (CC-PM), 3 (G-PM).

Regional fat-mass outcomes showed a similar descriptive pattern. In the CC-AM group, regional fat depots generally declined after the intervention, although the estimated changes were mostly small to modest. In the CC-PM group, reductions were observed in multiple regional fat compartments, including the upper limbs (mean change = −88.92 g, 95% CI, −155.88 to −21.96), thighs (mean change = −361.23 g, 95% CI, −677.90 to −44.56), trunk (mean change = −637.92 g, 95% CI, −983.50 to −292.35), android region (mean change = −131.23 g, 95% CI, −223.78 to −38.68), and gynoid region (mean change = −159.77 g, 95% CI, −279.57 to −39.96) (**Figure 5**). Change-score patterns again generally favored the CC-PM group, particularly for trunk and central fat measures (**Figure 6**).

Although the descriptive patterns tended to favor the CC-PM group over the CC-AM group, the estimated group × time effects for body weight, BMI, body fat percentage, total fat mass, and waist circumference were generally small to modest, with wide confidence intervals. The mean between-group differences in change were −0.48 kg (95% CI, −0.88 to 1.83) for body weight, 0.17 kg/m² (95% CI, −0.29 to 0.64) for BMI, 0.67% (95% CI, −0.43 to 1.78) for body fat percentage, 526.47 g (95% CI, −616.66 to 1669.60) for total fat mass, and −1.32 cm (95% CI, −3.48 to 0.83) for waist circumference. Given the modest sample size and the resulting uncertainty around these estimates, these between-group differences should be interpreted cautiously. The primary interpretation therefore focused on the descriptive contrast between the CC-AM and CC-PM groups, whereas the G-PM subgroup was retained only for completeness.

### Lifestyle-related factors during the intervention

To assess the potential influence of lifestyle-related factors during the intervention, physical activity, sleep quality, fatigue, and self-reported sleep–wake schedules were evaluated concurrently. Detailed IPAQ component scores for the CC-AM and CC-PM groups across baseline, week 2, week 4, week 6, and week 8 are presented in **Table 2**. In the CC-AM group, walking time increased numerically from 116.00 ± 121.69 min/week at baseline to 264.67 ± 226.27 min/week at week 8, and total MET increased from 1434.80 ± 1318.12 to 1726.73 ± 1433.78, whereas moderate activity, vigorous activity, and sitting time changed little overall. In the CC-PM group, moderate activity and total MET were numerically higher at week 2 and week 4 than at baseline, but these increases were not maintained through week 6 and week 8; vigorous activity also fluctuated across time points, including a temporary decrease at week 6. Overall, IPAQ-derived activity profiles showed only modest variation over time, and temporal patterns were broadly similar between the CC-AM and CC-PM groups. Because IPAQ assessments referred to activity during the preceding 7 days and therefore overlapped with the intervention period, these data should be interpreted cautiously and do not allow firm separation of prescribed exercise from habitual ayu ctivity outside the training sessions.

**Table 2:** IPAQ component scores across time points in the CC-AM and CC-PM groups (mean ± SD).

Group	IPAQ component	Baseline	Week 2	Week 4	Week 6	Week 8
CC-AM	Walking (min/week)	116.00 ± 121.69	150.33 ± 174.63	234.33 ± 218.44	159.67 ± 130.86	264.67 ± 226.27
	Moderate (min/week)	113.67 ± 152.09	159.33 ± 82.93	167.33 ± 114.55	179.33 ± 106.60	129.33 ± 155.45
	Vigorous (min/week)	74.67 ± 109.93	56.00 ± 89.19	86.00 ± 96.79	60.00 ± 90.71	42.00 ± 100.01
	Total MET	1434.80 ± 1318.12	1581.43 ± 1121.76	2130.63 ± 1206.98	1724.23 ± 1076.17	1726.73 ± 1433.78
	Sitting (h/day)	7.40 ± 0.83	7.13 ± 2.23	7.40 ± 1.92	7.53 ± 0.99	7.80 ± 0.94
CC-PM	Walking (min/week)	118.46 ± 135.04	151.92 ± 216.91	177.69 ± 126.63	177.31 ± 152.30	181.54 ± 164.41
	Moderate (min/week)	58.85 ± 83.37	233.08 ± 177.12	198.46 ± 165.02	184.62 ± 114.57	168.46 ± 124.02
	Vigorous (min/week)	91.15 ± 83.67	112.31 ± 161.41	88.46 ± 105.98	13.08 ± 27.50	60.00 ± 106.77
	Total MET	1355.54 ± 693.99	2332.12 ± 1699.69	2087.92 ± 861.29	1428.19 ± 864.03	1752.92 ± 966.85
	Sitting (h/day)	7.69 ± 0.95	7.85 ± 2.54	7.46 ± 2.37	7.85 ± 0.90	7.46 ± 1.13

Data are presented as mean ± SD. IPAQ, International Physical Activity Questionnaire; MET, metabolic equivalent of task. Group sizes: CC-AM (n = 15) and CC-PM (n = 13). A 2 (group) × 5 (activity) × 5 (time) repeated-measures ANOVA showed no substantial group × time interaction for IPAQ-derived activity components (F = 0.969, P = 0.428, partial η² = 0.036), indicating that temporal changes in self-reported physical activity patterns did not clearly differ between groups.

PSQI and FS-14 results suggested that sleep quality and fatigue varied somewhat during the intervention, but the overall changes were modest. Self-reported bedtime and wake-up time remained broadly stable from baseline to week 8 in both groups (**Table 3**). In the CC-AM group, mean bedtime changed from 00:14 ± 1:33 at baseline to 23:56 ± 1:27 at week 8, while mean wake-up time changed from 08:14 ± 1:06 to 08:35 ± 1:15. In the CC-PM group, mean bedtime was 00:13 ± 1:00 at baseline and 00:12 ± 1:05 at week 8, and mean wake-up time changed from 08:05 ± 1:05 to 08:27 ± 1:23. Estimated time in bed increased modestly in both groups, from 7.8 ± 1.4 to 8.6 ± 1.5 h in CC-AM and from 7.9 ± 1.1 to 8.3 ± 1.3 h in CC-PM. Overall, these descriptive data did not suggest major shifts in habitual sleep–wake schedules during the intervention. Similarly, PSQI and FS-14 results indicated that sleep quality and fatigue fluctuated to some extent during the intervention, but the overall changes were modest (P > 0.05). Self-reported bedtime and wake-up time showed no marked changes at baseline, week 4, and week 8 across groups, suggesting that habitual sleep–wake schedules remained broadly stable during the study period.

**Table 3:** Regional fat mass of each group before the intervention (mean ± SD).

Group	Upper limbs (g)	Thighs (g)	Trunk (g)	Android region(g)	Gynoid region(g)
CC-AM	3402.07 ± 3967.60	7860.13 ± 1676.75	10131.93 ± 2810.59	1504.47 ± 547.69	3907.07 ± 841.99
CC-PM	2413.85 ± 476.63	7433.15 ± 1715.98	10534.85 ± 3218.38	1598.15 ± 717.46	3636.77 ± 803.98
G-PM	5561.67 ± 1306.66	17813 ± 3056.70	22698 ± 2751.02	3248.33 ± 508.26	8142.67 ± 1234.20

### MEQ findings and correspondence with genotype

**Figure 7:**
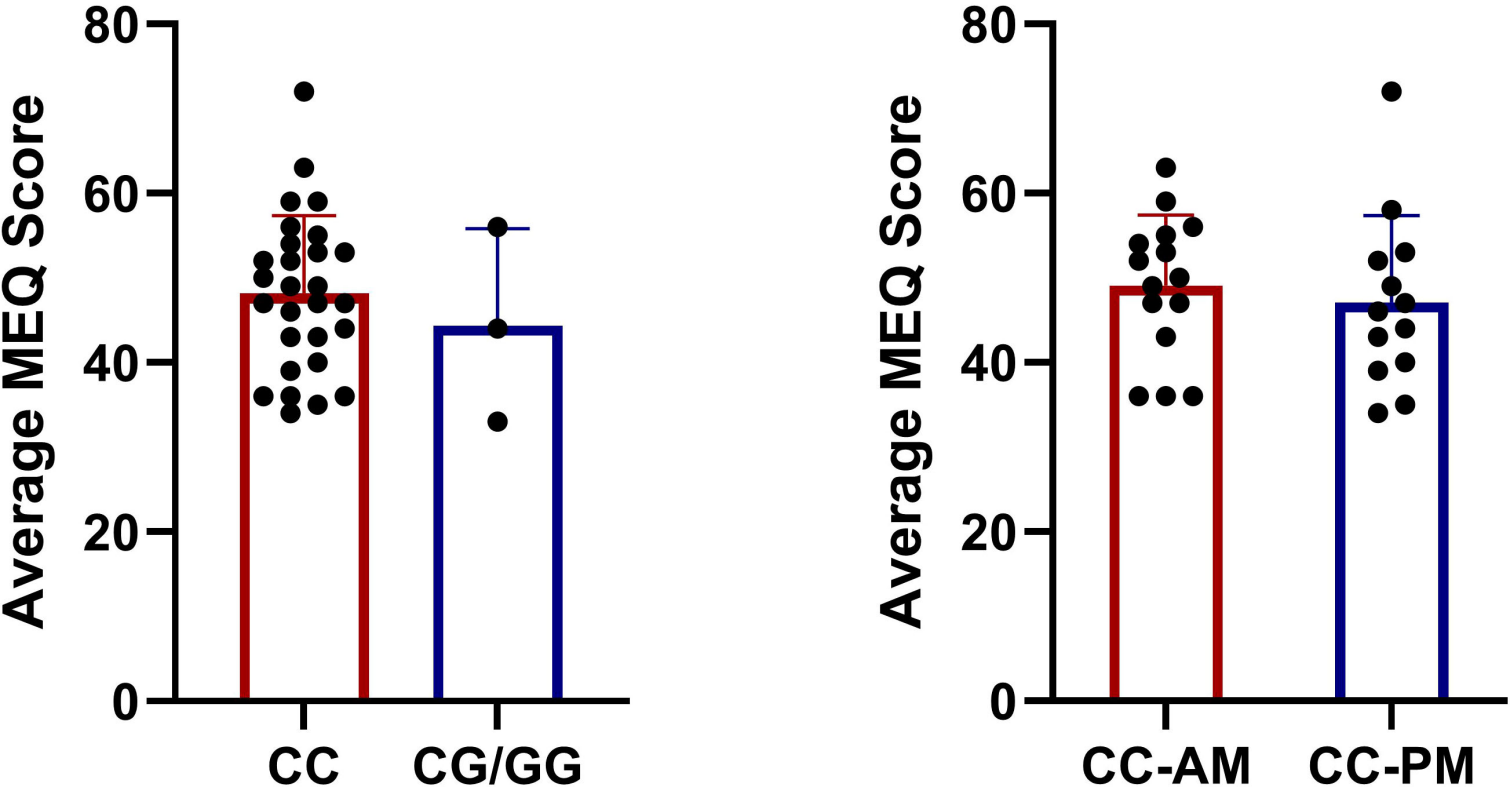
Baseline MEQ scores across groups. Left: MEQ scores by genotype group (CC homozygotes, n=28; G-allele carriers, n=3). Right: Comparison of MEQ scores between the CC-AM (n=15) and CC-PM (n=13) groups; bars represent group means; error bars indicate standard deviations (SD); jittered points show individual data.

Based on the MEQ results, participants were classified as morning type, intermediate type, or evening type. Baseline MEQ scores were 49.07 ± 8.38 in the CC-AM group and 47.08 ± 10.26 in the CC-PM group, corresponding to a mean difference of 1.99 points (95% CI, −5.25 to 9.23). MEQ scores were 48.14 ± 9.18 in CC homozygotes and 44.33 ± 11.50 in G-allele carriers, corresponding to a mean difference of 3.81 points (95% CI, −7.81 to 15.43). The distribution of chronotype categories across genotype groups is shown in **Figure 7**. Overall, behavioral chronotype did not show a clear correspondence with PER3 genotype in this sample.

### Exploratory analysis by MEQ-based chronotype tendency

To complement the genotype-based analyses, body-composition changes were also examined according to MEQ-based behavioral chronotype tendency (**Table 1**). Because only a small number of participants met the conventional MEQ criteria for definite morning or evening type, exploratory subgrouping was based on tertiles of the MEQ score distribution within the sample. Participants in the upper third of MEQ scores were categorized as the morning-leaning group (n = 10), those in the middle third as the intermediate group (n = 11), and those in the lower third as the evening-leaning group (n = 10).

Mean body weight change was −0.87 ± 1.52 kg in the morning-leaning group, −1.32 ± 1.93 kg in the intermediate group, and −0.91 ± 2.21 kg in the evening-leaning group. The corresponding mean differences were 0.45 kg (95% CI, −1.26 to 2.16) for morning-leaning versus intermediate, 0.04 kg (95% CI, −1.71 to 1.79) for morning-leaning versus evening-leaning, and −0.41 kg (95% CI, −2.12 to 1.30) for intermediate versus evening-leaning.

Mean body fat percentage change was −0.21% ± 0.62% in the morning-leaning group, −0.52% ± 1.23% in the intermediate group, and −0.94% ± 2.01% in the evening-leaning group. The corresponding mean differences were 0.31% (95% CI, −0.95 to 1.56), 0.73% (95% CI, −0.55 to 2.01), and 0.42% (95% CI, −0.83 to 1.68), respectively.

Mean total fat mass change was −389.70 ± 818.03 g in the morning-leaning group, −742.00 ± 1256.47 g in the intermediate group, and −1023.00 ± 2141.76 g in the evening-leaning group. The corresponding mean differences were 352.30 g (95% CI, −991.22 to 1695.82), 633.30 g (95% CI, −741.84 to 2008.44), and 281.00 g (95% CI, −1062.52 to 1624.52), respectively. Overall, the estimated differences across MEQ-based tendency groups were small to modest and imprecise, without a clear pattern suggesting meaningfully different body-composition responses to MICT according to behavioral chronotype tendency.

## Discussion

This exploratory study developed a genotype-based exercise-timing model using the PER3 rs228697 locus to examine the effects of 8 weeks of moderate-intensity continuous training (MICT) on body composition in overweight and obese university students. Across the overall sample, the pattern over time was consistent with reductions in body weight, BMI, waist circumference, body fat percentage, and total fat mass following the intervention, suggesting that 8 weeks of MICT may be beneficial for improving body composition in this population. In subgroup analyses, CC genotype carriers who trained in the afternoon (CC-PM) showed descriptively greater reductions across several adiposity-related outcomes than those who trained in the morning (CC-AM), whereas waist circumference showed the clearest change in the CC-AM group. However, the estimated between-group differences in change scores and the group × time interaction effects were modest and associated with wide confidence intervals. Taken together, these findings do not support the assumption that exercise timing aligned with PER3 rs228697 necessarily provides greater short-term body-composition benefits. Rather, within this exploratory sample and under the present MICT protocol, afternoon training among CC genotype carriers was associated with a more favorable descriptive pattern of change than morning training, although the uncertainty around the estimates precludes firm conclusions regarding genotype-based exercise-time matching based on PER3 rs228697 alone.

These findings suggest that the relationship between exercise timing and fat-loss outcomes may be more complex than a simple model of “better circadian alignment leads to better outcomes.” Circadian rhythm theory would predict that individuals with morning-type tendencies may respond more favorably to morning exercise, whereas those with evening-type tendencies may perform better or feel more comfortable exercising later in the day (Chtourou and Souissi, 2012b). In the present study, however, no clear advantage of genotype–timing alignment was observed. Instead, CC genotype carriers showed a descriptive tendency toward greater improvement when exercising in the afternoon. This pattern suggests that responses to exercise timing may depend not only on genotype-related circadian tendencies but also on actual behavioral rhythms, physiological responsiveness at different times of day, and individual differences in internal load during training.

Previous research on the effects of exercise timing on weight reduction has generated mixed findings, which adds context to the interpretation of the present results. In a long-term intervention study of overweight and obese adults, Willis et al. reported differences in weight-loss responses across exercise-timing groups; however, these effects were not consistent across all assessment time points, and no significant weight change was observed in the Early-EX group at the 3.5-month assessment (Willis et al., 2020b). This suggests that intervention duration may influence the relationship between exercise timing and weight-loss outcomes. In contrast, Creasy et al. and Mancilla et al. found that afternoon exercise may be more effective for reducing body weight, fat mass, and body fat percentage (Mancilla et al., 2021; Creasy et al., 2022). Taken together, these findings imply that the impact of exercise timing on fat-loss outcomes is unlikely to be uniform and may be influenced by participant characteristics, exercise modality, intervention duration, adherence, and circadian-related individual differences (Thun et al., 2015; Vitale and Weydahl, 2017; Facer-Childs et al., 2018). As noted in the review by Gabriel and Zierath, circadian rhythms clearly influence exercise performance and metabolism, but there is still no consensus on how exercise timing should be individualized to optimize metabolic health (Gabriel and Zierath, 2019). Against this background, the more favorable descriptive changes observed in the CC-PM group should not be interpreted as overturning current theory, but rather as an indication that exercise-timing effects may vary across individuals and may not be adequately captured by a single-locus matching model.

One possible explanation for the descriptively greater fat-loss response in the CC-PM group is that exercise performed at different times of day may impose different levels of internal training load, even when the external training load is standardized. In the present study, all groups received the same training frequency, duration, and relative intensity; therefore, the prescribed external load was broadly comparable across groups. However, perceived exertion, physiological stress, and metabolic responses may vary according to the time of day at which exercise is performed (Atkinson and Reilly, 1996; Callard et al., 2000; Guette et al., 2005; Teo et al., 2011). If participants exercise during a relatively non-preferred or less physiologically favorable time window, they may need to mobilize greater physiological resources to complete the same task, potentially resulting in a stronger internal training stimulus (Teo et al., 2011). Chtourou and Souissi proposed that training during periods of relatively lower physiological performance may not improve immediate performance, but may provide a greater adaptive challenge (Chtourou and Souissi, 2012b). Similarly, Anderson et al. suggested that training time, circadian preference, and PER3 genotype may influence exercise performance and perceived effort, with some individuals requiring greater effort to complete the same task during non-preferred times (Rae et al., 2015; Anderson et al., 2018b). In this context, the larger reductions observed descriptively in the CC-PM group may reflect differences in internal load rather than a direct benefit of genotype–timing mismatch itself. However, because internal training load was not objectively assessed in the present study, this interpretation remains speculative. Although the intervention lasted only 8 weeks, short-term differences in body-composition response may still emerge within this time frame. Thus, the more favorable descriptive changes observed in the CC-PM group may reflect an early response to the prescribed exercise timing rather than a definitive long-term benefit. The present study cannot determine whether these differences would persist over longer follow-up.

From the perspective of precision exercise prescription, another possible interpretation is that genotype–timing alignment may be more relevant to exercise tolerance, comfort, and subjective acceptability than to short-term fat loss per se (Küüsmaa et al., 2015). In other words, a time of day that feels more suitable for exercise may not necessarily be the one that produces the greatest short-term change in body composition. If aligned timing reduces perceived fatigue or physiological stress, the metabolic stimulus may also be lower under otherwise identical external loads. Conversely, if adherence and tolerance remain acceptable, exercise performed at a non-aligned time may elicit stronger fat mobilization or energy expenditure responses because of greater physiological challenge (Vitale et al., 2017). This interpretation also implies that in precision weight management, exercise-timing strategies should not be based solely on genotype-informed alignment, but should also account for training goals, individual tolerance, daily routines, and long-term adherence.

Another possible explanation for the present findings is non-exercise physical activity (NEPA). Previous studies have shown that structured exercise may alter habitual movement outside the exercise sessions, either through compensatory reductions or increases in daily activity (King et al., 2009; Pontzer, 2015; Melanson, 2017). Therefore, exercise timing may influence not only the response to the exercise bout itself, but also physical activity patterns and sedentary behavior during the remainder of the day, which may in turn affect total daily energy expenditure and body-composition change (King et al., 2009; Pontzer, 2015; Melanson, 2017). In the present study, questionnaire-based IPAQ component scores showed only modest variation over time, and temporal patterns were broadly similar between the CC-AM and CC-PM groups. However, because IPAQ assessments referred to activity during the preceding 7 days and therefore overlapped with the intervention period, these data cannot clearly separate prescribed exercise from habitual activity outside the supervised sessions. In addition, questionnaire-based assessment is not sensitive to subtle behavioral changes. Accordingly, the possibility that MICT influenced NEPA or sedentary behavior cannot be excluded. Future studies should use objective monitoring methods to clarify whether exercise timing influences NEPA and sedentary time (King et al., 2009).

It is also noteworthy that the MEQ results in this study did not show a clear correspondence between PER3 rs228697 genotype and behavioral circadian traits. This finding is consistent with the broader view that a single genetic locus is unlikely to explain an individual’s full morningness–eveningness phenotype (Archer et al., 2008; Jones et al., 2016). Although previous studies have suggested that the G allele of rs228697 may be associated with evening preference and the C allele with morning preference, actual circadian behavior is influenced by multiple factors, including sleep habits, school schedules, light exposure, social jet lag, and lifestyle. In the present study, self-reported bedtime and wake-up time recorded at baseline, week 4, and week 8 remained broadly stable in both groups, and estimated time in bed increased only modestly over the intervention. These descriptive findings do not suggest major shifts in habitual sleep–wake schedules during the study. However, because sleep timing was assessed by self-report and only at selected time points, these data could not fully capture day-to-day sleep–wake behavior or precisely determine the degree of alignment between actual circadian behavior and genotype-based classification (Lane et al., 2016). To provide complementary behavioral evidence, we also performed exploratory analyses based on the distribution of MEQ scores within the sample. Because only a limited number of participants met the conventional MEQ criteria for definite morning or evening type, participants were grouped into morning-leaning, intermediate, and evening-leaning categories according to the upper, middle, and lower thirds of the score distribution. Under this sample-based grouping approach, the estimated differences in changes in body weight, body fat percentage, and total fat mass were limited in magnitude and did not show a consistent pattern across groups; all corresponding confidence intervals included zero. These findings suggest that questionnaire-based behavioral chronotype tendency, at least as operationalized in the present sample, did not clearly distinguish responsiveness to MICT. Taken together, the present results indicate that neither PER3 rs228697 genotype nor MEQ-based behavioral chronotype tendency alone was sufficient to explain variation in body-composition response to the intervention. Future studies aiming to develop circadian-informed exercise-timing strategies should integrate genetic information with objective circadian markers and behavioral phenotyping in larger samples.

This study has several strengths, including the exploratory integration of circadian genetics and exercise timing in overweight/obese university students. However, the findings should be interpreted in light of several limitations. First, the total sample size was limited, and the G-PM subgroup was especially small (n = 3), which reduced the precision of subgroup estimates; therefore, findings involving G-allele carriers should be considered exploratory. Second, the intervention lasted only 8 weeks, so the study mainly reflects short-term changes in body composition and cannot determine whether the observed patterns would persist or contribute to long-term weight-loss maintenance. Third, although standardized nutritional counseling was provided, dietary intake and other potentially relevant behavioral factors, such as light exposure, sedentary behavior, and non-exercise physical activity, were not quantitatively controlled or objectively monitored; therefore, residual confounding and possible compensatory changes in habitual daily movement cannot be excluded. Fourth, circadian characterization remained limited: only one circadian-related locus (PER3 rs228697) was examined, and although self-reported bedtime and wake-up time were collected at baseline, week 4, and week 8, no objective or continuous circadian monitoring (e.g., actigraphy, dim-light melatonin onset, or core body temperature rhythm) was performed. In addition, because only a small number of participants met the conventional MEQ criteria for definite morning or evening type, the exploratory MEQ-based subgroup analysis relied on sample-based score ranking rather than standard categorical cutoffs. Finally, internal training load was not objectively assessed, so the possibility that time-of-day differences in perceived or physiological effort contributed to the observed descriptive patterns remains speculative. These factors may have limited the precision and generalizability of circadian classification and treatment-response interpretation in the present study.

## Conclusion

In this exploratory sample of overweight/obese university students, 8 weeks of moderate-intensity continuous training was associated with favorable changes in several body-composition outcomes. Within the exploratory exercise timing model based on PER3 rs228697, CC genotype carriers who trained in the afternoon showed descriptively greater improvements across several adiposity-related measures than those who trained in the morning; however, the between-group differences and group × time interaction estimates were small and imprecise, with wide confidence intervals. The results therefore do not support strong conclusions regarding genotype-based exercise-time matching based on PER3 rs228697 alone. Larger, well-controlled studies incorporating objective circadian phenotyping, internal load monitoring, and broader genetic stratification are needed to clarify the potential role of genotype-informed exercise timing in personalized weight management.

## Data Availability

The original contributions presented in the study are included in the article/Supplementary Material. Further inquiries can be directed to the corresponding author.
