# Supplementary material for: Afternoon exercise was associated with greater fat loss than morning exercise in overweight/obese adults: an exploratory study of PER3 rs228697 genotype matching
Source: Front Physiol. 2026 Jul 30;17:1872652. doi: 10.3389/fphys.2026.1872652 (PMC13467756; doi:10.3389/fphys.2026.1872652)
Supplement: Supplementary file 1 [file Table1.docx]

**Table S1:** Changes in body weight, body fat percentage, and fat mass by MEQ-defined chronotype groups

| **Outcome** | **Chronotype Group** | **n** | **Mean Change (SD)** | **95% CI for Mean Change** | **Group Comparison** | **Mean Difference (95% CI)** | **P value** |
| --- | --- | --- | --- | --- | --- | --- | --- |
| **Body weight (kg)** | MT | 10 | −0.87 (1.52) | −1.96 to 0.22 | MT vs NT | 0.45 (−1.26 to 2.16) | 0.596 |
|  | NT | 11 | −1.32 (1.93) | −2.62 to −0.02 | MT vs ET | 0.04 (−1.71 to 1.79) | 0.963 |
|  | ET | 10 | −0.91 (2.21) | −2.49 to 0.67 | NT vs ET | −0.41 (−2.12 to 1.30) | 0.629 |
| **Body fat percentage (%)** | MT | 10 | −0.21 (0.62) | −0.65 to 0.23 | MT vs NT | 0.31 (−0.95 to 1.56) | 0.619 |
|  | NT | 11 | −0.52 (1.23) | −1.35 to 0.31 | MT vs ET | 0.73 (−0.55 to 2.01) | 0.254 |
|  | ET | 10 | −0.94 (2.01) | −2.38 to 0.50 | NT vs ET | 0.42 (−0.83 to 1.68) | 0.497 |
| **Fat mass (g)** | MT | 10 | −389.70 (818.03) | −974.89 to 195.49 | MT vs NT | 352.30 (−991.22 to 1695.82) | 0.595 |
|  | NT | 11 | −742.00 (1256.47) | −1586.11 to 102.11 | MT vs ET | 633.30 (−741.84 to 2008.44) | 0.354 |
|  | ET | 10 | −1023.00 (2141.76) | −2555.12 to 509.12 | NT vs ET | 281.00 (−1062.52 to 1624.52) | 0.672 |

Note: MT, morning type; NT, intermediate type; ET, evening type. Chronotype groups were defined based on MEQ tertiles. Mean differences and 95% CIs are from Tukey post hoc comparisons following one-way ANOVA. All confidence intervals included zero, indicating that the observed differences were compatible with sampling variability.

Table S2: Self-reported bedtime and wake-up time at baseline, week 4, and week 8 in the CC-AM and CC-PM groups

| **Group** | **Time Point** | **Bedtime (mean ± SD)** | **Wake-up Time (mean ± SD)** | **Time in Bed (h, mean ± SD)** |
| --- | --- | --- | --- | --- |
| **CC-AM** | Baseline | 00:14 ± 1:33 | 08:14 ± 1:06 | 7.8 ± 1.4 |
|  | Week 4 | 23:52 ± 1:12 | 08:13 ± 0:54 | 8.2 ± 1.2 |
|  | Week 8 | 23:56 ± 1:27 | 08:35 ± 1:15 | 8.6 ± 1.5 |
| **CC-PM** | Baseline | 00:13 ± 1:00 | 08:05 ± 1:05 | 7.9 ± 1.1 |
|  | Week 4 | 23:59 ± 1:13 | 08:24 ± 1:32 | 8.4 ± 1.4 |
|  | Week 8 | 00:12 ± 1:05 | 08:27 ± 1:23 | 8.3 ± 1.3 |

Note: Times are presented in 24-hour format (mean ± SD). Time in bed was calculated as wake-up time minus bedtime. Group sizes: CC-AM (n=15), CC-PM (n=13). Due to missing or uninterpretable entries in the original dataset, not all participants contributed data at every time point; the displayed means are based on available data for each time point within each group. Between-group differences at each time point were small and not analyzed inferentially, consistent with the descriptive and exploratory nature of this assessment.
